# Alarmin Function of Galectin-9 in Murine Respiratory Tularemia

**DOI:** 10.1371/journal.pone.0123573

**Published:** 2015-04-21

**Authors:** Anthony L. Steichen, Tanner J. Simonson, Sharon L. Salmon, Dennis W. Metzger, Bibhuti B. Mishra, Jyotika Sharma

**Affiliations:** 1 Department of Basic Sciences, University of North Dakota School of Medicine and Health Sciences, Grand Forks, North Dakota, United States of America; 2 Center for Immunology and Microbial Disease, Albany Medical College, Albany, New York, United States of America; Public Health Research Institute at RBHS, UNITED STATES

## Abstract

Sepsis is a complex immune disorder that is characterized by systemic hyperinflammation. Alarmins, which are multifunctional endogenous factors, have been implicated in exacerbation of inflammation in many immune disorders including sepsis. Here we show that Galectin-9, a host endogenous β-galactoside binding lectin, functions as an alarmin capable of mediating inflammatory response during sepsis resulting from pulmonary infection with *Francisella novicida*, a Gram negative bacterial pathogen. Our results show that this galectin is upregulated and is likely released during tissue damage in the lungs of *F*. *novicida* infected septic mice. In vitro, purified recombinant galectin-9 exacerbated *F*. *novicida*-induced production of the inflammatory mediators by macrophages and neutrophils. Concomitantly, Galectin-9 deficient (Gal-9^-/-^) mice exhibited improved lung pathology, reduced cell death and reduced leukocyte infiltration, particularly neutrophils, in their lungs. This positively correlated with overall improved survival of *F*. *novicida* infected Gal-9^-/-^ mice as compared to their wild-type counterparts. Collectively, these findings suggest that galectin-9 functions as a novel alarmin by augmenting the inflammatory response in sepsis development during pulmonary *F*. *novicida* infection.

## Introduction

Systemic hyperinflammation is the underlying cause of many immune inflammatory diseases, including sepsis. Despite more than three decades of active research, severe sepsis and septic shock remain major healthcare challenges with a mortality rate of 20–50% and no effective therapy [1]. During sepsis, overwhelming and sustained release of pro- as well as anti-inflammatory cytokines, termed cytokine storm, causes extensive tissue damage and wide-spread cell death, eventually resulting in death. Despite the identification of cytokine circuitry as major determinants of mortality, pro-inflammatory cytokine blockade has been ineffective as therapy for sepsis. This indicates the involvement of additional mediators that are likely acting as regulators and/or perpetuators of this hyper inflammatory response.

Alarmins are evolutionarily conserved endogenous molecules that perform homeostatic functions when contained within cellular compartments [2]. However, under pathological conditions, these molecules can be released either passively from dead cells or actively via non-classical secretion pathways [3]. Once in the extracellular milieu, they exhibit immune modulatory properties such as induction of pro-inflammatory cytokines, immune cell chemotaxis, and regulation of cell death [2]. In fact, a sustained and excessive release of alarmins has been shown to contribute to pathogenesis of several sterile as well as infectious inflammatory conditions [4,5]. Pertaining to their ability to impact innate immune cells such as macrophages, dendritic cells and neutrophils, alarmins also represent a crucial link between innate and adaptive immune responses, and hence an attractive therapeutic target for complex disorders such as sepsis.

Francisella is a highly virulent bacterial pathogen that causes an acute lethal disease called tularemia in humans and mice. Although there are strain-dependent differences in the initial mechanisms involved [6,7], our studies have shown that pulmonary infection of mice with fully virulent *Francisella tularensis* as well as the murine model organism *F*. *novicida* (F.n.) leads to development of severe sepsis characterized by hyperinflammation, T cell depletion, and extensive cell death in systemic organs [8–10]. As pulmonary infections are a major cause of sepsis [11], we are using a murine inhalation model of F.n. infection to understand the mechanism/s responsible for pulmonary infection-induced sepsis development. A recent report of an F.n. outbreak in a correctional facility suggests that this strain may be more virulent to humans than initially surmised, supporting its relevance as a model strain to understand pathogenesis [12]. Furthermore, studies from our and other laboratories have shown that extensive tissue damage and wide-spread cell death is a hallmark of Francisella infection, regardless of the bacterial strain [9,13,14]. Additionally, our studies show that Francisella infected macrophages are defective in clearance of dead cells, a process termed efferocytosis, leading to accumulation of these dead cells and their progression to secondary necrosis [15]. It is thus likely that alarmins released from these dead or dying cells contribute to the inflammatory response culminating in sepsis development during respiratory infection with Francisella. We and others have reported an alarmin-mediated regulation of the inflammatory response during pulmonary infections [16–18]. However, in a complex immune disorder like sepsis which is an interplay of several host immune pathways such as the coagulation system, complement cascade and even the autonomic nervous system [19], several alarmins may be involved at the intersections of these pathways. Thus, identification of additional alarmins will present therapeutic targets that may have more tangible translational potential when used in combination.

Galectins, mammalian β-galactoside binding lectins, are emerging as potent immune regulators in a variety of pathological processes including inflammation, autoimmunity, fibrosis, and cancer [20,21]. Curiously, some galectins (galectin-1 and -3) have been shown to be secreted in the extracellular milieu via a non-classical ER/Golgi-independent pathway, where they exert immune modulating effects on immune cells. This is a characteristic feature of alarmins [22,23]. However the contribution of galectins as alarmins to sepsis development is poorly understood. In this study we have investigated the role of galectin-9, in the F.n. induced inflammatory response and sepsis. Our analyses of galectin-9 expression, distribution, immune modulation and comparison of disease progression in F.n. infected galectin-9 sufficient and—deficient mice show that galectin-9 acts as an alarmin to exacerbate the inflammatory response in Francisella infection induced sepsis development.

## Materials and Methods

### Ethics Statement

The animal usage protocols were approved by the Institutional Animal Care and Usage Committee at the University of North Dakota (protocol no. 1108–3). All procedures strictly followed the institutional and federal guidelines and all efforts were made to minimize the animal suffering.

### Bacterial strains and Mice

The F.n. strain U112 was grown on Trypticase Soy Agar (TSA) medium supplemented with L-cysteine at 37°C. After overnight growth, the bacteria were harvested and suspended in a freezing medium (250 mM sucrose, 10 mM sodium phosphate pH 7.2 and 5 mM glutamic acid). Aliquots of the stocks were frozen at -80°C for further use. The animal usage protocols were approved by the Institutional Animal Care and Usage Committee at the University of North Dakota. All procedures strictly followed the institutional and federal guidelines and all efforts were made to minimize animal suffering. In vivo experiments were performed using 6–8 wk old female C57Bl/6 wild-type and Gal-9 ^-/-^ mice. Gal-9 ^-/-^ mice were kindly provided by Dr. Judy M. Teale, University of Texas at San Antonio (initially obtained from the Consortium of Functional Glycomics, Scripps, La Jolla) as F4 and were back-crossed for additional 6 generations. Sex- and age-matched Gal-9^+/+^ mice with the same genetic background were used as controls.

### Antibodies and Reagents

All reagents were purchased from Sigma-Aldrich unless otherwise indicated. For detection of galectin-9 by immunofluorescence (IF) staining, a purified rat galectin-9 antibody (Abcam, San Diego, CA) followed by Alexa-546 conjugated chicken anti-rat antibody (Molecular Probes, OR) was used. A rat anti-mouse CD11b antibody conjugated to PE (BD Pharmingen) and a purified rat anti-mouse Ly-6G (Clone Accurate Chemical, Westbury, NY, USA) followed by chicken anti-rat Alexa488 (Molecular Probes, OR) were used for detection of activated neutrophils by IF staining. For flow cytometry Pacific Blue anti-mouse CD11b and APC anti-mouse Ly6G (Clone 1A8) antibodies (Biolegend, San Diego, CA) were used. The terminal deoxyribonucleotidyl transferase-mediated triphosphate (dUTP)-biotin nick end labeling (TUNEL) staining kit was purchased from Chemicon International, CA. Purified recombinant galectin-9 was purchased from R&D Systems, MN. The endotoxin level was <1.0 EU per μg of protein. Ultrapure *E*. *coli* endotoxin was purchased from Invivogen and lactose was purchased from Sigma. For detection of reactive oxygen species, Fc OxyBURST assay reagent was purchased from Molecular Probes, Eugene, OR. Mouse IL-6 ELISA kits (BD OptEIA) were from BD Biosciences, San Diego, CA.

### Infection of Mice, survival and bacterial burden

Mice were anaesthetized using a mixture of ketamine HCL and xylazine (30mg/ml ketamine, 4 mg/ml xylazine in PBS) and were infected intranasally with 50–70 CFUs of the F.n. strain U112 in 20 μl of PBS or with 20 μl of PBS alone. The mice were monitored daily. The survival of infected mice (15 mice each WT and Gal-9^-/-^ in 3 independent experiments) was recorded for up to 2 weeks post-infection (p.i.). The death was recorded as tularemia induced mortality. Mice displaying severe signs of distress (labored breathing, non-responsiveness to cage tapping, failure of grooming and severe eye discharge) were humanely sacrificed by injecting a mixture of ketamine (90–120mg/kg) and xylazine (10mg/kg) followed by cervical dislocation. In some experiments, the mice were euthanized at indicated times p.i. and blood, lungs, liver and spleen were aseptically harvested. The organs were homogenized aseptically in cold PBS with Complete protease inhibitor cocktail (Roche Diagnostics, Germany). For the bacterial burden analyses, the homogenates and blood were serially diluted in PBS and plated on TSA. CFU counts per mouse were calculated after incubating the plates at 37°C overnight.

### Quantitative real-time PCR

Lungs from infected and mock control mice at various times p.i. were removed immediately after perfusion and total RNA was extracted using Trizol reagent (Invitrogen) according to the manufacturer’s instructions. Real-time PCR analysis of the samples was performed using SYBR green (Applied Biosystems, CA, USA) as described by us [17]. Transcript levels of the housekeeping ribosomal 18S and galectin-9 genes were measured in each sample by PCR amplification using specific primers: 18S (forward) 5'-CATGTGGTGTTGAGGAAAGCA-3' and (reverse) 5'-GTCGTGGGTTCTGCATGATG-3'; Gal-9 (forward) 5'- TCAAGGTGATGGTGAACAAGAAA-3’ and (reverse) 5’- GATGGTGTCCACGAGGTGGTA -3’. The target gene expression levels were normalized to those of the house keeping 18S gene in the same sample. Expression of galectin-9 in infected samples was determined as fold change over that in control samples calculated by using the formula 2^−(ΔΔCt)^.

### Histological and Immunofluorescence staining

For histological and immunofluorescence staining, frozen lung tissues were processed as previously described [8]. Lung cryosections thus obtained were stained with hematoxylin and eosin (H&E) for pathological analysis, or for the detection of galectin-9 by immunofluorescence staining, as previously described [24]. Semi-quantitative histopathology was performed as described previously in [25]. For the detection of cell death, TUNEL method was used according to manufacturer’s instructions (Chemicon International, CA). The images were acquired using a Nikon Eclipse fluorescent microscope with an attached SPOT II digital camera. The images were processed and analyzed using Adobe Photoshop 7.0 software (Adobe, Mountain view, CA).

### Flow cytometry

Lungs were harvested from the infected and mock control mice at 3 days p.i. (dpi) after perfusion with PBS and were treated with collagenase to obtain single cell suspensions as previously described [8,9]. Cells were then co-stained with anti-CD11b and-Ly6G antibodies. FlowJo (Tree Star) software was used to analyze data.

### Multi-analyte profile analysis

The lung homogenates were prepared as described for the bacterial burden analysis above and were centrifuged at 2000 x g for 15 min to clear cellular debris. The supernatants were immediately frozen at -80°C. The biomarker levels in lung homogenates were determined commercially by Myriad Rules-based Medicine (Austin, TX, USA) utilizing a multiplexed flow-based system: Mouse MAP (Multi-Analyte Profiles) analysis technology.

### Neutrophil and macrophage activation

Cells were isolated from the peritoneal cavities of naïve C57BL/6 mice 12–14h after intraperitoneal injection of sterile 4% thioglycollate. Non-adherent neutrophils were plated at the density of 1x10^6^ cells and were infected with wild-type F.n. strain U112 at MOI 50 with or without pretreatment of the cells with 15μg/ml of purified recombinant galectin-9. Cells stimulated with galectin-9 alone or with 10ng/ml of phorbol myristate acetate (PMA) served as controls. One hour after stimulation, production of reactive oxygen species (ROS) was measured in the cells by flow cytometry using Fc OxyBURST reagent according to the manufacturer’s instructions. A minimum of 20,000 events were read for each sample and all the cells fluorescing positive in FITC channel (excitation and emission maxima of ~490 nm and ~520 nm, respectively) were gated to obtain the percentage of ROS producing cells.

Bone marrow was isolated from wild-type and Gal-9^-/-^ mice and the cells were differentiated to macrophages as previously described [26]. On day 6 of culture, the cells were plated at 8×10^4^ cells / well in 96-well flat-bottom plates and were stimulated with galectin-9 with or without 25mM lactose. As a control for endotoxin contamination, recombinant galectin-9 or UltraPure LPS boiled for 45 min at 100°C were used to stimulate cells. Culture supernatants were collected 24h after stimulation and IL-6 levels were measured by ELISA according to the manufacturer’s instructions (BD OptEIA, BD Biosciences).

### Statistical Analysis

Statistical analysis of survival studies was performed by Kaplan Meir log-rank test; bacterial burdens by non-parametric Mann-Whitney Test. All other statistical analyses were performed using two-way ANOVA with Tukey post-test.

## Results

### Galectin-9 is upregulated and released during the septic phase of F.n. infection

**Fig 1:**
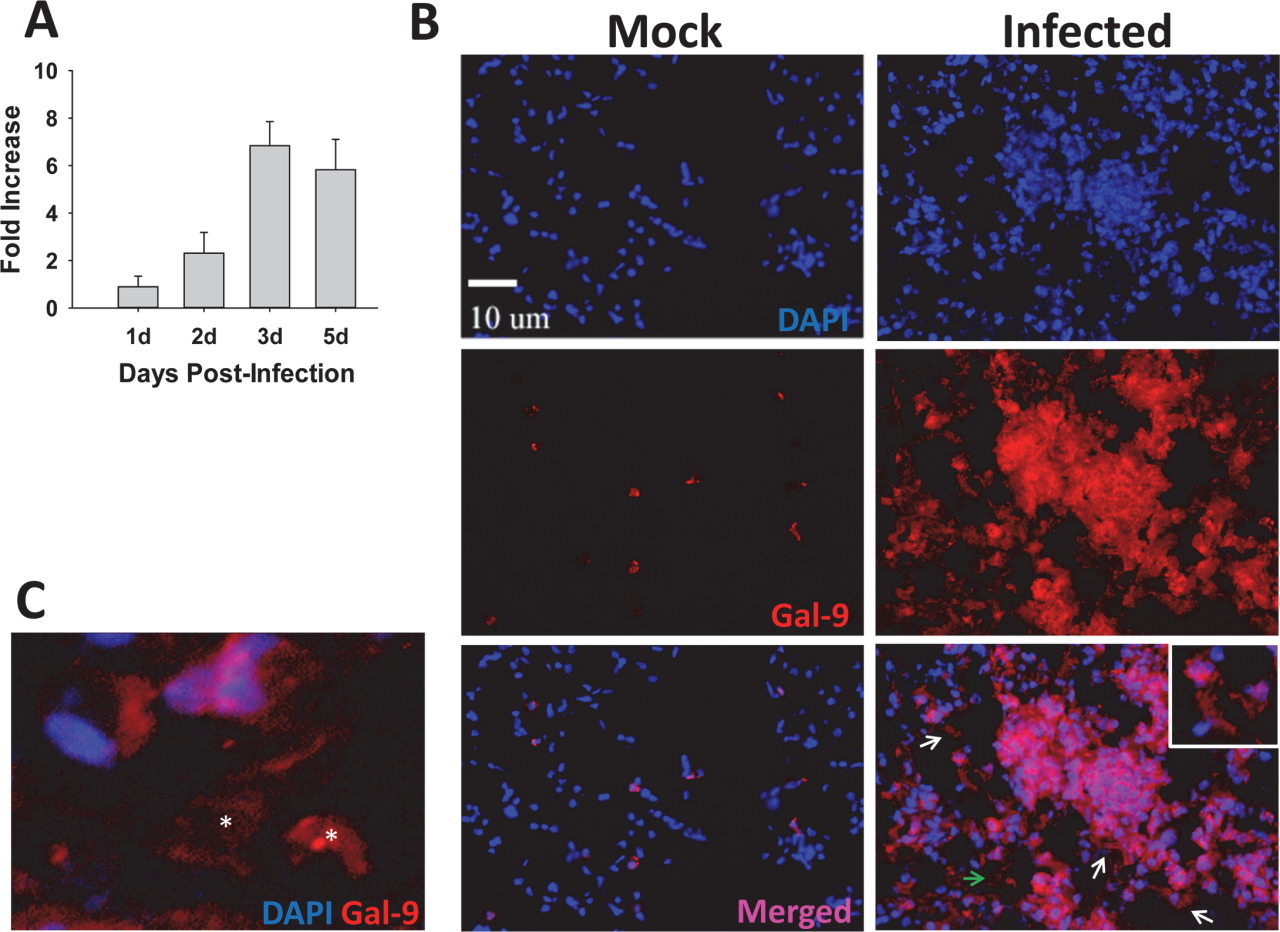
Galectin-9 is upregulated and released in lungs of mice during respiratory *F*. *novicida* (F.n.) infection. **(A)** Total RNA was extracted by Trizol method from lungs harvested at the indicated times after infection with the F.n. strain U112. The mRNA levels of galectin-9 were analyzed by real-time PCR as described in Materials and Methods and are expressed as fold increase over the levels in mock control mice. Data shown are the averages of 3–4 mice per group. **(B)** In-situ IF staining of frozen lung sections from mock control and U112 infected mice harvested at 3 dpi. The sections were stained for galectin-9 (red) using a purified rat galectin-9 antibody followed by Alexa-546 conjugated chicken anti-rat antibody. Nuclei (blue) were stained with 4’6’ diamidino-2-phenylindol-dilactate (DAPI). Magnification X 200. Inset depicts possible extracellular galectin-9 in infected mouse lungs. The area indicated by green arrow has been enlarged and shown in **(C)**. Asterisks depict galectin-9 present in cell-free areas.

We and others have previously shown that extensive cell death is a hallmark of pulmonary Francisella infection, regardless of the bacterial strain used (8, 9, 21–23). Factors released from dead or dying cells, termed as alarmins, have been shown to exhibit inflammatory, chemoattractant and immune activating properties in various pathological conditions. To test our hypothesis that galectin-9 functions as an alarmin, we first analyzed the expression and distribution of galectin-9 in the lungs of mice undergoing pulmonary infection with F.n. As shown in Fig 1A, galectin-9 transcript levels progressively increased in the lungs of infected mice and reached the highest levels at 3dpi. This increase in galectin-9 expression at 3 dpi is consistent with the appearance of other sepsis features (extensive cell death, hyperinflammatory response, increased vascular injury) at this time, as reported in our previous studies with F.n. as well as the fully virulent *F*. *tularensis* [8,9]. Immunofluorescence (IF) analysis of galectin-9 protein expression in cryosections of the lungs harvested at this septic phase showed a low basal level of galectin-9 in mock control mice inoculated with PBS alone (Fig 1B). F.n. infected mice, on the other hand, exhibited very high galectin-9 expression in their lungs. Furthermore, galectin-9 could be detected in cell-free areas, especially in the lesions with massive cellular infiltration, indicating that it is likely released extracellularly (Fig 1B lower panel and inset). Together, these data suggested that galectin-9 likely exhibits a characteristic alarmin property of extracellular release during septic phase of pulmonary F.n. infection.

### Galectin-9 deficiency ameliorates lung pathology in F.n. infected mice

**Fig 2:**
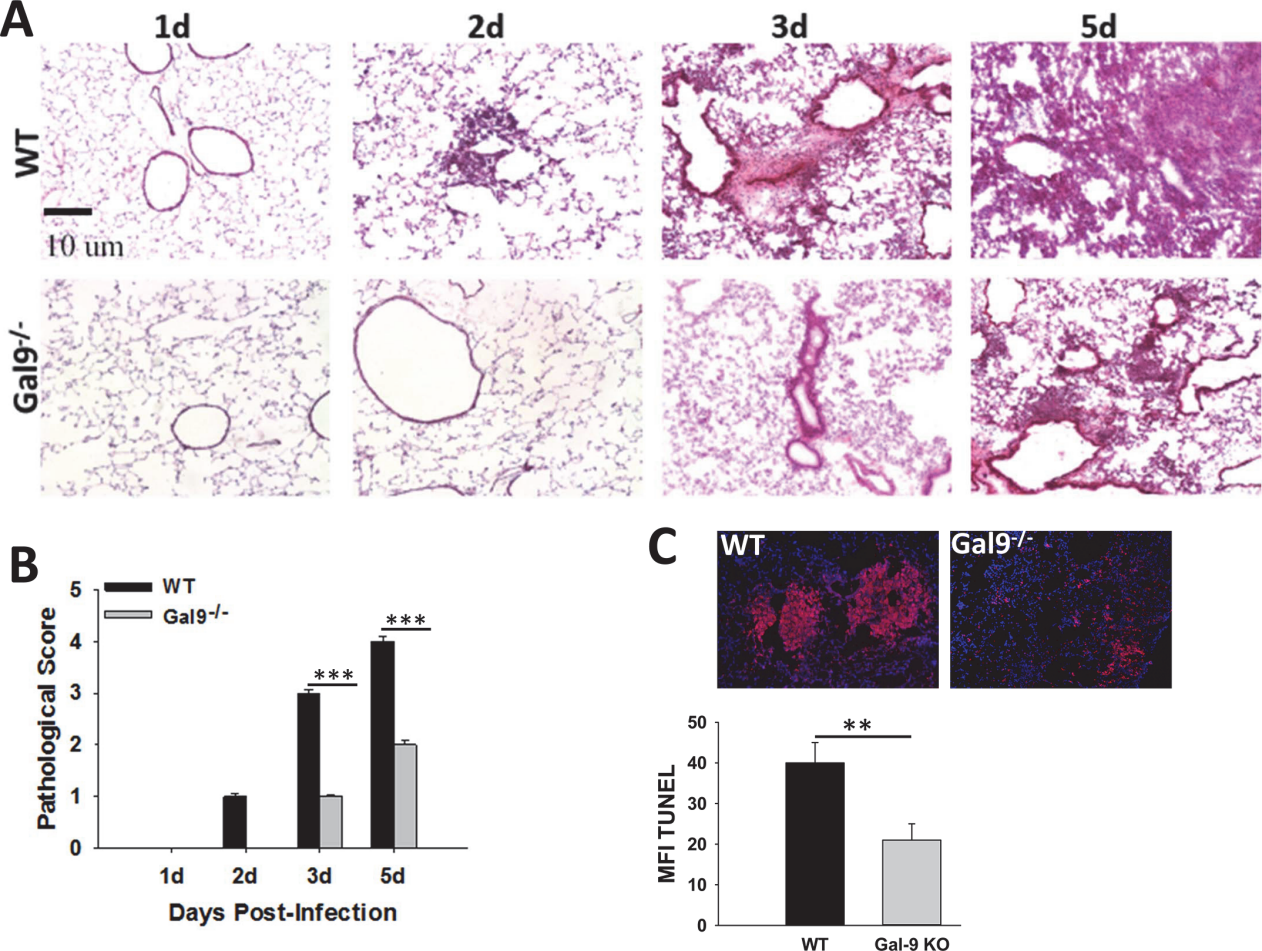
Gal-9^-/-^ mice exhibit improved lung pathology and reduced TUNEL staining indicative of cell death upon pulmonary F.n. infection. **(A)** The lungs from F.n. infected wild-type (WT) or Gal-9^-/-^ mice were harvested at indicated times post-infection, embedded in optimal-cutting-temperature (OCT) compound, and sectioned as described in Materials and Methods. The frozen sections were stained with Hematoxylin and Eosin (H&E). The images obtained are representatives of three experiments performed with 3 mice per group in each experiment. Magnification, ×200. **(B)** H&E sections were scored in blinded fashion according to the following scoring scale: 0, no inflammatory cells (macrophages or neutrophils) present in section; 1, <5% of section infiltrated by inflammatory cells; 2, 5–10% of section infiltrated; 3, 20% of section infiltrate; and 4, >20% of section infiltrated. **(C)** Frozen lung sections from F.n. infected WT or Gal-9^-/-^ mice were processed for in-situ TUNEL staining for detection of DNA fragmentation (red) in nuclei. Nuclei (blue) were stained with 4′,6′-diamidino-2-phenylindole dilactate. Bar graph shows Mean fluorescence intensity (MFI) quantified using Image J software. Magnification, ×100.

In order to assess the role of galectin-9 in overall disease severity during tularemia, we next examined H&E stained lung cryosections from F.n. infected wild-type (WT) and Gal-9^-/-^ mice. Consistent with our previous studies, a progressive increase in cellular infiltration was observed in the F.n. infected WT mice with extensive pathology, severe bronchopneumonia and massive cell death, occurring in the center of large granuloma-like areas of infiltration at 3 dpi (Fig 2A upper panel). The lungs of Gal-9^-/-^ mice, on the other hand, showed a substantial delay in the cellular infiltration with moderate peribronchial and perivascular infiltration by 3dpi (Fig 2A lower panel). Although these mice displayed an increased cellular infiltration at 5dpi, the lesions appeared much smaller in the lungs of Gal-9^-/-^ mice as compared to those in the WT mice. Concomitantly, the Gal-9^-/-^ mice exhibited significantly lower pathological score in comparison with their WT counterparts at all the time points tested (Fig 2B). Mock infected WT and Gal-9^-/-^ mice exhibited similar normal lung architecture with minimal cellular infiltration and clear air spaces (data not shown). As alarmins are released passively from dead cells, we analyzed the extent of cell death in the lungs of infected WT and Gal-9^-/-^ mice by TUNEL assay. As shown in Fig 2C, septic lungs of F.n. infected WT mice showed extensive cell death, indicated by a large number of TUNEL positive cells within perivascular and peribronchial lesions. In contrast, infected Gal-9^-/-^ mice exhibited significantly fewer TUNEL positive cells in their lungs (Fig 2C). The improved lung architecture and reduced cell death in the absence of galectin-9 indicates a pathological role of this lectin during pulmonary Francisella infection.

### Galectin-9 regulates cellular infiltration and inflammatory response in vivo during F.n. infection

**Fig 3:**
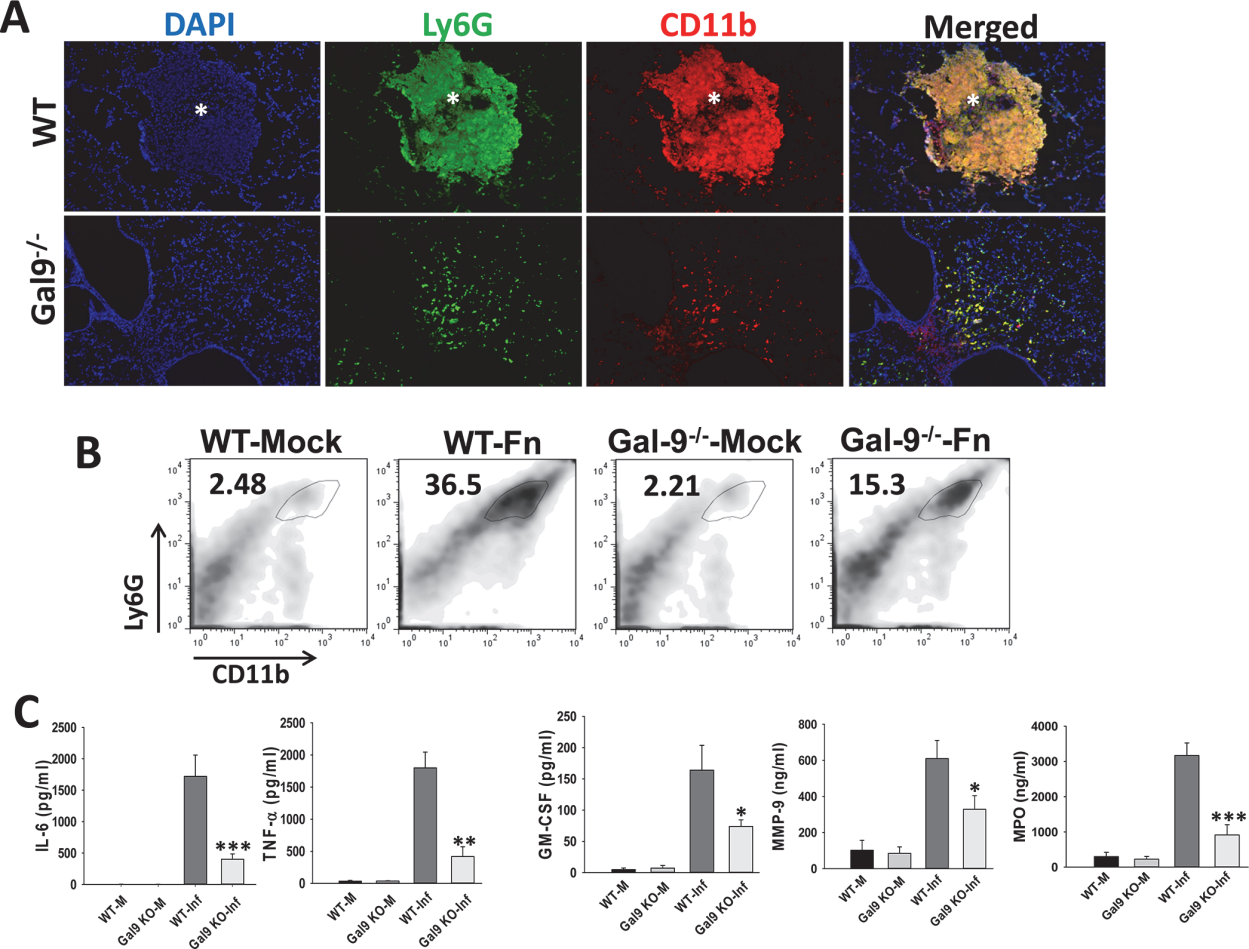
Gal-9^-/-^ mice display reduced accumulation of neutrophils and reduced inflammatory mediators in lungs during F.n. infection. **(A)** Frozen sections of lungs harvested at 3 dpi from F.n. infected WT or Gal-9^-/-^ mice were co-stained with antibodies against myeloid cell markers CD11b (red) and Ly6G (green). A high co-expression of both markers is depicted by yellow color and indicates neutrophils. Nuclei (blue) were stained with 4’6’ diamidino-2-phenylindol-dilactate (DAPI). Magnification X 200. Asterisks depict lesions in the lungs. **(B)** Flow cytometry analysis of neutrophils in mock control and F.n. infected WT and Gal-9^-/-^ (WT-Fn and Gal-9^-/-^-Fn) mice. Total lungs cells were isolated from mice by collagenase treatment at 3 dpi as described in Methods. The cells were stained with anti-Ly6G-APC and anti-CD11b-Pacific Blue antibodies as markers for neutrophils. The plots are representative of three mice per group in 3 independent experiments. **(C)** Lungs from mock infected and F.n. infected WT or Gal-9^-/-^ mice were harvested at 3dpi, homogenized with protease inhibitors in PBS and analyzed commercially for rodent multi-analyte profiles (Myriad Rules-Based Medicine, Austin, TX). Levels of inflammatory cytokines and neutrophil markers in lung homogenates are shown. Results shown are from 3–4 mice per group from 3 different experiments. MMP-9; matrix metalloproteinase 9, MPO; myeloperoxidase. * *p*<0.05; ** *p*<0.005; *** *p*<0.001. Comparisons were made between infected WT and Gal-9^-/-^ groups.

Alarmins exert their immune stimulatory effects by mediating immune cell infiltration directly or indirectly, and by predisposing the cells to produce increased levels of inflammatory mediators in response to infection. We hypothesized that; similar to the function of alarmins, increased expression and extracellular localization of galectin-9 may be contributing directly or indirectly to the increased cellular infiltration and hyperinflammatory response observed during pulmonary Francisella infection. Previous studies from our and other laboratories have shown that neutrophils are the predominant cell type infiltrating in F.n. infected WT mice [8,9,27,28]. In order to compare neutrophil infiltration in the lungs of Gal-9^-/-^ and WT mice infected with F.n., IF staining and flow cytometry was performed. Consistent with our previous studies, lungs of the infected WT mice showed extensive infiltration of CD11b+ Ly6G+ neutrophils that accumulated largely in the lesions (Fig 3A and 3B). The numbers of Ly6G+CD11b+ cells in Gal-9^-/-^ mice, on the other hand, were significantly reduced (Fig 3A and 3B). These results suggested a role of galectin-9 in regulation of neutrophil accumulation in the lungs of mice during pulmonary F.n. infection. Furthermore, analysis of the levels of inflammatory cytokines and neutrophil-associated mediators in the lungs revealed significantly reduced levels of TNF-α, IL-6, myeloperoxidase (MPO), matrix metalloproteinase-9 (MMP-9) and GM-CSF, in the infected Gal-9^-/-^ mice (Fig 3C). Together, these results suggested a putative role of galectin-9 in neutrophil accumulation and associated inflammation.

### Galectin-9 exacerbates F.n. infection induced inflammatory response in vitro

**Fig 4:**
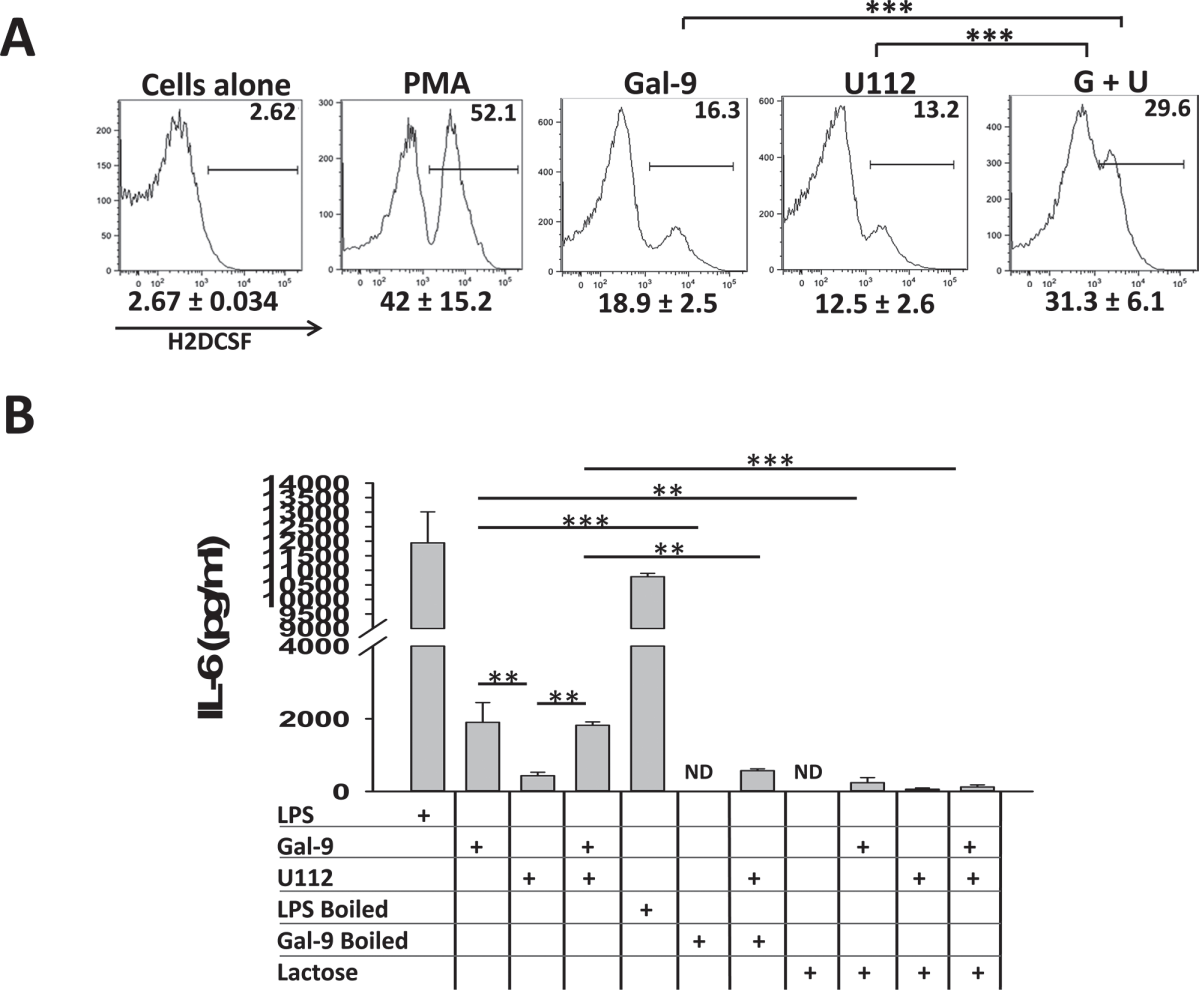
Galectin-9 regulates F.n. infection induced inflammatory response in vitro. **(A)** Peritoneal neutrophils were isolated from mice 12–14h after injection with 4% thioglycollate and were stimulated with F.n. at an MOI 50 with or without pre-treatment with purified recombinant galectin-9 (15μg/ml). Stimulation with galectin-9 alone or phorbol myristate acetate (PMA, 10ng/ml) was used as a control. Production of reactive oxygen species (ROS) was measured one hour post-stimulation by flow-cytometry using Fc-OxyBURST dye following the manufacturer’s instructions. Numbers below the plots depict average percent of ROS positive cells from 3 independent experiments. Plots from a representative of these 3 independent experiments are shown. **(B)** Bone marrow derived macrophages (BMDMs) from C57Bl/6 wild-type mice were stimulated with F.n. Strain U112 at an MOI of 50 with or without pretreatment with 15μg/ml of purified recombinant galectin-9. UltraPure E.coli LPS (10ng/ml) and galectin-9 with and without heat-denaturation (boiled at 100°C for 45 min) as well as competitive inhibition with lactose (25mM) were used as controls to test the specificity of galectin-9 effect, as described in methods. Culture supernatants were collected 24h after infection and the amount of IL-6 was measured by ELISA. The data shown is average of three independent experiments. Statistical analysis between the data sets was performed by Two-way ANOVA with Tukey post-test where * *p*<0.05; ** *p*<0.005; *** *p*<0.001.

To examine the direct effect of galectin-9 on activation of neutrophils and macrophages, two cell types playing a predominant role in tularemia pathogenesis, we next carried out in vitro stimulation of these cells with recombinant galectin-9 protein with or without F.n. infection. While stimulation with purified galectin-9 or U112 infection alone moderately induced ROS production in peritoneal neutrophils, pre-treatment of neutrophils with this lectin induced an increased amount of ROS in response to F.n. infection, which was significantly higher than that elicited by galectin-9 or F.n. infection alone (Fig 4A). Stimulation of bone marrow derived macrophages (BMDMs) with galectin-9 alone or galectin-9 and U112 together elicited a significantly higher IL-6 production than U112 alone. (Fig 4B). This immune stimulatory effect of galectin-9 was abolished upon heat-denaturation or by addition of 25mM lactose to the cultures, confirming the specificity of this activity. These observations indicated that while galectin-9 by itself can stimulates myeloid cells, particularly macrophages, it can also augment Francisella infection-induced myeloid cell activation which likely has implications in exacerbation of inflammation culminating in sepsis development during this infection.

### Gal9^-/-^ mice show improved survival following F.n. infection

**Fig 5:**
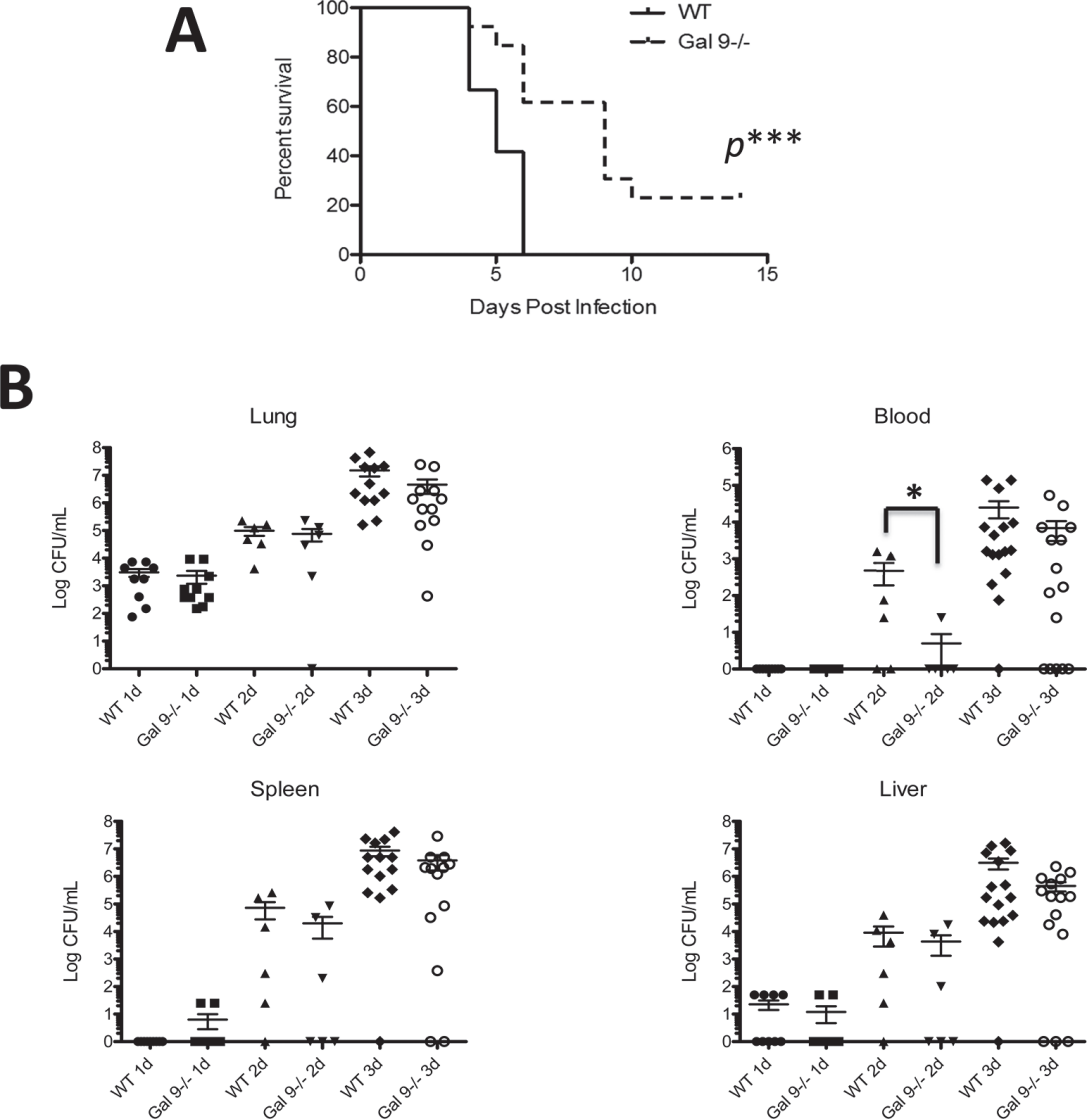
Gal-9^-/-^ mice show improved survival during pulmonary F.n. infection. **(A)** Fifteen each C57Bl/6 WT and Gal-9^-/-^ mice in 3 separate experiments (5 mice per experiment) were inoculated intranasally with F.n and were monitored daily for 2 weeks. The improved survival of Gal-9^-/-^ mice compared to WT mice was statistically significant, as determined by Kaplan-Meier log-rank analysis (*P* value*** = 0.0003). **(B)** Bacterial burdens in lungs, blood, spleen and liver harvested from F.n. infected WT and Gal-9^-/-^ mice at indicated times post-infection. Lung, liver and spleen homogenates prepared as described in Materials and Methods and blood were serially diluted and plated on TSA plates to enumerate bacterial burdens. Each symbol on the plots represents one mouse and data is from 2–3 independent experiments.

In order to correlate the improved lung pathology and reduced inflammatory responses with the disease outcome, overall disease severity and survival was compared in WT and Gal-9^-/-^ mice infected with F.n. In the infected WT mice, visible signs of disease started to appear by 3 dpi which typically included piloerection, hunched gait, lethargy, and eye discharge. All of these mice succumbed to infection by day 6 p.i. (Fig 5A). By contrast Gal-9^-/-^ mice exhibited delayed appearance of disease symptoms and showed significantly improved survival as compared to the infected wild-type mice (Fig 5A). The improved survival of Gal-9^-/-^ mice, however, did not correlate with bacterial burdens in their lungs, spleen and liver since similar bacterial load was observed in WT and KO mice (Fig 5B). Intriguingly, Gal-9^-/-^ mice exhibited significantly lower bacterial counts in their blood at 2dpi than their WT counterparts at that time point indicating a delayed development of bacteremia in the absence of galectin-9. However, the bacterial burden in blood was similar in infected WT and Gal-9^-/-^ mice at later time points indicating that once hematogenous, bacteria replicated at similar rates in both strains.

## Discussion

In this study we examined the role of galectin-9, a mammalian β-galactoside binding lectin, in the development of sepsis during pulmonary infection with *F*. *novicida*. We report the upregulation and extracellular release of galectin-9 during F.n. infection and its ability to amplify Francisella infection-induced inflammatory response as well as concomitant improved disease severity of Gal-9^-/-^ mice. These functions of galectin-9 are all consistent with characteristic properties of alarmins that have been described to play a pathological role in perpetuation of inflammation.

Sepsis is characterized by a complex, systemic inflammatory response to a traumatic or infectious insult, such as F.n. infection in the current study. Mechanisms of innate immune activation in sepsis are no longer thought be induced exclusively by components of pathogen origin (pathogen associated molecular patterns, PAMPs). Alarmins, that are multifunctional host proteins, have been shown to regulate inflammatory response in many pathological conditions including sepsis, upon their release from dead/dying host cells [29–31]. High Mobility Group Box1 (HMGB-1), S100 family of proteins, and heat shock proteins are among the most well-characterized alarmins to date [3], although the list of alarmins has continued to grow over the past decade [32]. Indeed, in a complex immune disorder like sepsis, it is likely that several alarmins are involved at the intersections of various pathways. Our current study shows that galectin-9, a host lectin, plays a pathogenic role as an alarmin to exacerbate the inflammatory response during pulmonary infection with Francisella and contributes to sepsis development. Identification of novel alarmins such as galectin-9 may aide in understanding this complex disorder and may present additional targets for effective therapeutics.

Galectins are ubiquitously expressed β-galactose binding lectins. These constitute the only soluble mammalian lectins which are either passively released by dying cells or are actively secreted by inflammatory cells through a non-classical secretory pathway [33]. Although several galectins have been detected in the extracellular milieu [34], the precise mechanism of their secretion remains to be determined, since they lack a classical “leader” sequence. Galectins play homeostatic roles in regulation of cell cycle and apoptosis, phagosome formation, and stabilization of intracellular signaling when contained in intracellular compartments [35]. Some of these galectins such as galectin-1 and -3 exhibit inflammatory and T cell apoptotic activities upon extracellular release [36]. Moreover, the extracellular release of some galectins seems to correlate with the virulence of invading pathogen [23,37] as well as influences immune responses through chemotaxis and activation of innate immune cells [33,36]. Despite the progress in our understanding of the inflammatory properties of galectins, the role of these lectins as alarmins in the development of sepsis is not defined. Our results described here show, for the first time that galectin-9, mostly studied for its function as modulator of T cell immunity, is likely released extracellularly and modulates innate immune responses by affecting immune cell infiltration and activation during a septic infection by F.n.

Although speculative at this stage, the reduced number of leukocytes in F.n. infected Gal-9^-/-^ mice suggests that this lectin directly or indirectly affects cellular infiltration/accumulation in sepsis. To the best of our knowledge, there are no reports examining the impact of galectin-9 on innate cell infiltration during an acute pneumonic infection although, human galectin-9 has been identified as a potent eosinophil chemoattarctant with implications in allergic airway inflammation [38]. Galectin-9 has also been shown to induce IL-8 production by bronchial epithelial cells in cystic fibrosis, influencing neutrophil infiltration and early neutrophil-dominated inflammation in the cystic fibrosis lung [39]. Reduced levels of neutrophil chemoattractant and activation markers in Gal-9^-/-^ mice in our studies suggest that this lectin may exert its effect on cellular infiltration indirectly by modulating the levels of these chemokines. This observation is in line with the role of the prototypic alarmin HMGB1 in neutrophil migration by regulating the levels of chemoattractants such as IL-8 [40]. However it is also possible that galectin-9 influences neutrophil infiltration directly by binding to its receptor T cell Ig and mucin domain–containing molecule-3 (TIM-3) which was recently reported to be expressed by neutrophils [41]. Other proinflammatory alarmins such as S100 proteins have also been shown to directly promote migration of myeloid cells by binding to their cell surface receptors [42]. Further studies are needed to test these hypotheses. Notwithstanding the mechanism, the reduced neutrophil accumulation in F.n. infected Gal-9^-/-^ animals indicates a likely chemotactic function of this lectin in F.n. induced sepsis.

The role of galectin-9 in regulating T helper-1 responses via its interaction with TIM-3 has been extensively studied. Galectin-9 interaction with TIM-3 on T cells results in cell death causing down regulation of pro-inflammatory Th1 responses implicated in pathology of autoimmune diseases such as EAE and GVDH [43–45]. However, its role in myeloid cell activation is much less defined. Recent studies have reported involvement of TIM-3/galectin-9 interaction in activation of neutrophils and macrophages [41,46]. Another study showed galectin-9 induced secretion of pro-inflammatory cytokines from monocytes and macrophages, that could amplify immunopathology associated with HCV infection [47]. Additionally, the effects of galectin-9 on activation and maturation of antigen presenting cells have been shown [48–50]. More relevant to our study, a recent study reported that galectin-9 mediates potentiation of TLR-ligation induced TNF-Α and IL-6 secretion from microglia [51]. This is similar to our observation of galectin-9 mediated potentiation of *F*. *novicida* induced inflammatory response. In our studies, consistent with the property of a typical alarmin, galectin-9 exhibited immune activating properties such as stimulation of the oxidative burst in neutrophils and inflammatory cytokine production in macrophages. Importantly, this lectin was able to augment F.n. infection induced inflammatory responses from neutrophils as well as macrophages, which can have important implications under in vivo conditions. Consistent with this pro-inflammatory activity of galectin-9, Gal-9^-/-^ animals exhibited a reduction in the levels of inflammatory cytokines such as TNF-α, IL-6 and IL-1 during F.n. infection. Studies from our laboratory have shown that pulmonary infection with F.n., as well as *F*. *tularensis*, results in extensive cell death [15]. It is likely that during pulmonary Francisella infection, galectin-9 released from dead cells primes the myeloid cells to produce heightened levels of inflammatory mediators in response to the bacteria. Furthermore, since galectin-9 can be upregulated and released from cells upon exposure to pro-inflammatory stimuli such as cytokines and TLR ligands [52], the activated cells likely release more galectin-9 resulting in a positive feed-back loop causing further tissue damage and ultimately organ failure, characteristic of sepsis. This is also supported by our unpublished observations showing that bone marrow derived macrophages from Gal-9^-/-^ mice show impaired activation upon stimulation with TLR-4 ligand LPS. This immune stimulatory activity of galectin-9 in the context of Francisella infection is in line with a previous study showing the role of the prototype alarmin HMGB1 in promoting the inflammatory response of monocytes elicited by external stimuli [53].

As a result of mitigated inflammatory responses and tissue pathology, the survival of F.n. infected Gal-9^-/-^ mice was significantly improved as compared to the WT mice which succumbed to infection by day 5–6. Interestingly, we observed similar bacterial burdens in the lungs of F.n. infected Gal-9^-/-^ and wild-type mice. However, the bacteremia was delayed in Gal-9^-/-^ mice with significantly lower burden in blood at 2dpi but the difference at later points as was not statistically significant as compared to the WT mice. Galectin-9 has been shown to mediate phagocytosis of *P*. *aeruginosa* by neutrophils thus contributing to bacterial clearance [41]. In contrast, a similar bacterial burden in WT and Gal-9^-/-^ mice in our study indicates that galectin-9 does not play a role in F.n. clearance. Because of high bacterial burden, a majority of Gal-9^-/-^ mice ultimately succumbed to infection. These results are similar to our recently published studies of galectin-3 function in F.n. induced sepsis. In this scenario a combinatorial approach using blockade of galectin-3 and galectin-9 along with antibiotics could potentially treat Francisella infection induced sepsis. Additionally, in order to address any possible synergistic effect of galectin-3 and -9 (since we have found that these are the only two galectins, out of nine tested by us, which are upregulated and are likely released in Francisella infected lungs), we have already generated galectin-3/galectin-9 double knock-out mice and are currently analyzing them for sepsis development during bacterial infection. In light of a similar alarmin-like function of these galectins, the double knock-out mice may exhibit further improvement in the disease outcome.

Together our findings indicate that galectin-9 plays a pathogenic role as an alarmin to exacerbate the inflammatory response during pulmonary infection with Francisella and contributes to sepsis development. Galectin-9 thus may represent a potential target for treatment of sepsis during this infection.
